# Practical Notes and Formulæ

**Published:** 1886-03-20

**Authors:** 


### PRACTICAL NOTES AND FORMULÆ.

**Tonic and Alterative.**—Anæmia and chlorotic patients will fatten and thrive woderfully on the following mixture (by Goodell); but it should not be given for any longer period than two weeks at a time:

℞. Hydrargyum chloridi corosivi.........  ......gr. i–ij,

Liquoris arseniosi chloridi....................f. ʒ i,

Tincture ferri chloridi; } aa..............f. ʒ iv,

Acidi Hydrochlorini diluti, }

Syrupi.................................f. ℥ v.

M. Signa: One (1) dessertspoonful in a wineglassful of water after each meal.

**Turpine.**—The bi-hydrate of turpentine has received the name turpine. It is said to possess the medicinal qualities of turpentine without its disagreeable odor and flavor. It acts on the bronchial mucous membrane, the kidneys and nervous system. It is given in doses from 3 to 5 drops.

**Mucilage.**—J. C. (Philadelphia, Pa.)—Mr. T. W. Watkins, says the Therapeutic Gazette, states that a mucilage of acacia, which will not spoil, may be made as follows:

℞. Oil of gaultheria..............................*m* xv,
Calcium phosphate...........................sufficient.
Water............  ..........................℥ viij,
Acacia.......................................℥ iv.

Triturate the oil of wintergreen with about one drachm of the phosphate of calcium and afterwards the water, and filter. Then use the filtrate to make a mucilage with the acacia.—*Druggists Circular and Chemical Gazette.*

**Pimple Ointment.**—(Druggists Circular and Chemical Gazette)

℞. White precipitate..........................gr. x,
Cold cream...................................℥ i

Mix. Apply every morning.

**Chilblain Remedy.**—(*Ibid*) W. E. T., Lowell, Mass., sends this formula:

℞. Essence sassafras..............................℥ i,

Tincture iodine................................dr. ss.

One or two applications will cure any ordinary case in a few hours. Can also be used in erysipelas and glanular swellings.

**Hypodermic Quinine Solution.**—(Drug. Cir.) Prof. S. S. Burt says, in the Quarterly Bulletin Clinical Society, that he has found the following a satisfactory solution of quinine for hypodermic use:

℞. Quinine bisulphate..........................grs. lx,
Boric acid..........................grs. ij,
Morphine sulphate..........................grs. $\frac{1}{4}$,
Distilled water..........................℥ j.

S. For use subcutaneously; 1 dr. represents 7½ grains quinine.

**Carson's Paint.**—(*Ibid.*)

℞. Croton oil..........................ʒ ij,

Ether..........................ʒ iv,

Comp. tinct. iodine..........................ʒ x.

Applied with camel's hair brush, to produce mild pustular eruption.

**Iodine in the Treatment of Chronic Rheumatism.**—M. A. Fort (Union Medicale) gives the following formula;

℞. Potassium iodide..........................ʒ v,

Potassium bromide..........................gr. lxxv,

Syrup of gentian..........................℥ xviij,

Tincture of iodine..........................dr. xx.

A tablespoonful to be taken, morning and evening, in chronic articular rheumatism, and the affected joints to be painted with tincture of iodine.

**Superior Tonic Mixture.**—A writer in Eastern Med. Journal mentions the following as an admirable tonic, and specially useful in diabetis. The following is the formula, which any druggist can prepare:

℞ Ferri sulphus..........................ʒ ss,
Soda phosphate..........................ʒ iij,
Sulph. quinine..........................grs. lxvi,
Ar. sulph. acid..........................q. s.,
Strychnia..........................grs. iv,
Sach. alba..........................℥ vij.

Dissolve iron ℥ ss in boiling water, dissolve soda in ℥ j boiling water.

Mix the two solutions and wash until the iron is tasteless. Dissolve the quinine in ar. sulph. acid, add ʒ ij of water. Precipitate with aquæ ammonia, after which carefully wash.

Dissolve the sulph. iron and quinine thus obtained, and strychnia in dil. phos. acid, then add sugar and dissolve without heat. Bottle and keep from the air and light.

Dose: 20 to 30 drops.

**Syrup of White Pine.**—(National Druggist.)

℞. Fluid extract of white pine bark..........................fl. ℥ 2,
Simple syrup..........................fl. ℥ 14.

